# When depression threatens retention: psychological ownership and work engagement as protective factors for healthcare workers

**DOI:** 10.1108/JHOM-10-2025-0670

**Published:** 2026-05-18

**Authors:** Krista Howard, Stefanie Sliger, Yejun (John) Zhang, Stephanie Dailey, Jérémy E. Wilson-Lemoine

**Affiliations:** Department of Psychology, Texas State University, San Marcos, Texas, USA; Department of Management, Texas State University, San Marcos, Texas, USA; Department of Communication Studies, Texas State University, San Marcos, Texas, USA

**Keywords:** Depression, Healthcare workers, Turnover intention, Psychological ownership, Work engagement

## Abstract

**Purpose:**

Depression is a prevalent mental health challenge that impairs employees’ ability to function at work and may shorten how long they stay with an organization. Healthcare workers (HCWs) face heightened vulnerability due to emotional strain, long hours and job demands. Drawing on the job demands-resources model, this study examined whether psychological ownership and work engagement could help lessen the negative impact of depression on HCWs’ plans to remain with their employer.

**Design/methodology/approach:**

We collected data from 199 US HCWs, measuring depressive symptoms, psychological ownership, work engagement and the likelihood of staying at their organization for five years.

**Findings:**

About one in five participants (22.1%) screened positive for major depressive disorder (MDD) and were less likely to intend to stay with their work organization long term. However, when psychological ownership and work engagement were included, depression was no longer a significant predictor of turnover intention.

**Practical implications:**

These findings suggest that interventions that foster a sense of ownership and engagement may help healthcare organizations retain valuable staff, even among those experiencing depressive symptoms.

**Originality/value:**

This study is among the first to show that depression does not inevitably undermine healthcare workers’ retention intentions when psychological ownership and work engagement are present. By integrating the job demands-resources framework with positive psychological resources, the study advances theory and offers actionable pathways for organizations to support workforce stability despite mental health challenges.

Depression is known as one of the most common mental disorders in the world, reportedly affecting more than 280 million people globally (Kessler, 1994; Liu *et al*., 2024). The Diagnostic and Statistical Manual of Mental Disorders-fifth edition (DSM-5) criteria for the diagnosis of major depressive disorder (MDD) must include a presence of either a persistent depressed mood or a lack of interest or pleasure in almost all activities for a period of at least two weeks. In addition to at least one of those two, the diagnostic criteria mandate the presence of at least four other relevant symptoms: sudden weight loss or gain, loss of appetite, insomnia or fatigue, reduced ability to think or concentrate and psychomotor agitation, among others (American Psychiatric Association, 2013). Problematically, depression affects mood, emotions, engagement and cognitive abilities, often hindering individuals’ ability to function effectively in both their personal and professional domains.

Organizational research has shown that depression is not only due to individual or personal factors, but it can also be caused by workplace factors (for reviews and meta-analyses, see Khalid and Syed, 2024; Mikkelsen *et al*., 2021; Pega *et al*., 2021; Sussell *et al*., 2025). Scholars have identified a broad set of stressors macro- (i.e. societal; e.g. economic crisis), meso- (i.e. organizational; e.g. harmful workplace interactions) and micro-levels (i.e. individual; e.g. personality) factors that can affect depression (Khalid and Syed, 2024; Mikkelsen *et al*., 2021). Steadman and Taskila (2015) classify the work-related impacts of depression into three overarching domains: (1) *getting to work*, which is impeded by symptoms such as poor sleep, low motivation and anhedonia; (2) *doing the job*, which is compromised by low enthusiasm and difficulty concentrating; and (3) *working with others*, which may be disrupted by social withdrawal or intolerance of noise and interaction. These symptom clusters collectively impair job performance and elevate turnover risk. The specificities of the healthcare sector (e.g. work overload, long hours and lack of control) add additional sources of stressors that impact healthcare workers’ (HCWs) mental health (Sussell *et al*., 2025). However, measures exist to mitigate the impact of these stressors, including interventions (Aust *et al*., 2024), changes in work design (e.g. staffing, scheduling; van Kraaij *et al*., 2025) or shifts in organizational priorities (e.g. consideration of well-being and safety, Amoadu *et al*., 2023) and practices (e.g. greater emphasis on bottom-up communication, Amoadu *et al*., 2023). Nonetheless, when depression is not addressed, it can have a devastating impact on both the employees and the organization (Harvey *et al*., 2017; Khalid and Syed, 2024; WHO, 2022). Because depression compromises mood, motivation and concentration – factors that are critical to effectiveness in the workplace – it is no surprise that this mental health disorder is associated with workplace turnover (Hankin *et al*., 1998; Pang *et al*., 2020; Steadman and Taskila, 2015). Recent longitudinal evidence from the healthcare sector showed that depressive symptoms predict turnover intentions (Zhang *et al*., 2024). Indeed, a meta-analysis of 46,513 individuals confirmed that there is strong and consistent evidence that depressive symptoms negatively impact employment outcomes, including turnover intention (Steadman and Taskila, 2015), defined as the conscious and deliberate decision to leave an organization (Tett and Meyer, 1993). The adverse effects of depression on employment outcomes are well-documented. For example, research investigating the long-term effects of depression found that employed individuals experiencing depression were six times more likely to be unemployed and four times more likely to have experienced job turnover at a 6-month follow-up, when compared to their nondepressed counterparts, indicating these individuals either voluntarily chose to leave their job or were involuntarily terminated during this relatively short timeframe (Lerner *et al*., 2004). Although turnover intention has been used as a proxy for turnover (Hom *et al*., 2017), scholars have argued these concepts should not be used interchangeably (Lee *et al*., 2017), and the focus has more recently been on why people stay, rather than why people leave (Hom *et al*., 2017; Lee *et al*., 2017; Sánchez-Cardona *et al.*, 2023; for an example in the healthcare sector, see Hendrickson *et al*., 2022). We framed our study in line with these developments by exploring organizational longevity (i.e. intention to stay) rather than turnover intention.

Because healthcare workers routinely face high job strain – stemming from long hours, emotional demands and limited organizational support – they are especially vulnerable to depression and its associated consequences for employment longevity. A recent study found that 25% of nurses and 24% of physicians experience depression, indicating its potential impact on workplace longevity among healthcare workers (Olaya *et al*., 2021). At the same time, healthcare systems around the world are facing growing workforce shortages, making it increasingly important to understand not only how depression affects HCWs’ retention, but also which factors may potentially account for these effects. The current study examines this issue, focusing on psychological ownership and work engagement as potential protective factors that may account for the negative relationship between depression and workplace longevity in the healthcare context.

## Depression and turnover in the healthcare profession

The healthcare profession as a whole is extremely demanding and emotionally taxing. HCWs constantly face emotional and physical demands, work abnormally long hours and are exposed to high-stakes, life-or-death situations. This can compromise their physical and mental health and limit time for personal and family life. The global shortage of HCWs further exacerbates these challenges, as existing staff must absorb heavier workloads and meet heightened productivity expectations to offset staffing deficits (Jamaludin and Binti Zulkifli, 2025). This increased level of stress also increases the likelihood of employee burnout, which has been found to be the single strongest professional factor associated with depression (Fond *et al*., 2022). For example, studies have found that HCWs are more likely to experience depressive symptoms than the general population as a result of the demanding nature of their work (Belete and Anbesaw, 2022). A recent review highlights that the European healthcare sector is going through a crisis due to both distress and high turnover among HCWs (Almeida-Meza *et al*., 2025).

Although COVID-19 likely contributed to a spike in depressive symptoms among HCWs (Lavell *et al*., 2025; Wang and Welbourne, 2024), the prevalence of MDD in this population long predates the global crisis. Even before 2020, a substantial body of literature documented elevated rates of depressive symptoms among HCWs. For example, a 2012 study by Letvak *et al*. found that healthcare workers had more than double the rate of depressive symptoms (18%) compared to the general population (8%). Mata *et al*. (2015) documented a 0.5% annual increase in HCW depression rates. To potentially shed some light on more recent depression prevalence in HCWs, Fond *et al.* (2022) suggest that roughly one-third of HCWs suffer from depression, regardless of their role within the healthcare industry. This prevalence of depression in HCWs can have a significant impact on turnover intention within the industry. Post-pandemic reviews continue to identify psychological distress associated with negative organizational outcomes, including intentions to leave and workforce retention (Almeida-Meza *et al*., 2025; Jachmann *et al*., 2025). Given that depression is a well-established predictor of turnover intention, these prevalence rates highlight the urgent need to understand the mechanisms that contribute to both attrition and retention in this critical workforce.

The effects of depression on employee turnover within the healthcare industry are staggering. Pang *et al*. (2020) found that turnover intention was 280–460% higher in HCWs with moderate to severe depressive symptoms, when compared to other HCWs without such symptoms, underscoring the powerful role of depression in driving employee turnover within the healthcare industry. In addition to depression, the same conditions that fuel burnout – long shifts, overwork, emotional and physical exhaustion, and other workplace factors – also contribute to high turnover rates (Jamaludin and Binti Zulkifli, 2025). An umbrella review of high-income healthcare and welfare sectors showed that turnover is influenced by a mix of factors, including job characteristics (Wynendaele *et al*., 2025). A recent meat-analysis among nurses worldwide found that turnover intention is high, with two in five nurses who intend to leave (Mafula *et al*., 2025). High levels of turnover are also present among physicians (Wang *et al*., 2025). Global turnover rates among HCWs are stated to be between 15 and 27%, with recent US figures approximating 18.4% (Duffield *et al*., 2014; Nursing Solutions, Inc., 2024).

There are many costs of HCW turnover: monetary, organizational and intellectual. Financially, the recruitment, onboarding, and training of replacements are costly. For example, according to Nursing Solutions, Inc. (2024), the price of employee turnover for a staff nurse is around $56,300, highlighting this costliness and implying even higher expenses for HCWs turnover in more specialized positions. Razmpour *et al*. (2026) presented an even higher figure, stating that the turnover cost per nurse was $85,498.

Organizationally, HCW turnover creates staffing gaps that overburden remaining employees, lowering efficiency and productivity. Intellectually, HCW turnover represents a loss of expertise, experience and potentially a loss of specialized care (Jamaludin and Binti Zulkifli, 2025). These combined effects highlight the urgency of developing evidence-based strategies to reduce turnover intention and retain skilled HCWs.

## Seeking to account for HCW turnover

Although the job demands-resources (JD-R) model was first established to investigate characteristics of a job that could affect burnout via two different processes, namely job demands and job resources (Demerouti *et al*., 2001), the model has greatly evolved in the past 25 years. The JD-R theory now considers these processes as dynamic and reciprocal (Bakker and Demerouti, 2017). It describes dynamic demand-resource processes through which employees are not only influenced by the demands and resources of their job, but can also influence these demands and resources via job crafting (i.e. proactive changes employees can make to their work to make it more meaningful, such as learning a new skill; Bakker and Demerouti, 2017). The JD-R theory has also integrated the concept of work engagement, which is described as an important component that leads to greater proactive behaviors and job performance (Bakker and Demerouti, 2017). The model also highlights that these demands and resources can come from different levels (e.g. team, leader and organizational) and that these demands and resources interact with employees' levels of well-being or ill-being (Bakker *et al*., 2023). As such, when employees experience high levels of ill-being (e.g. depression), they may experience more strongly the negative impact of job demands. In the present study, we draw on the JD-R theory by considering psychological ownership and work engagement as resources and depression as an indicator of ill-being. We aim to investigate the relationship between depression and organizational longevity in HCWs and to assess if psychological ownership and/or work engagement account for the relationship between depression and organizational longevity.

### Psychological ownership

Psychological ownership refers to an individual’s sense of possessiveness toward an organization or aspect of their work, which reflects identity, belonging, and perceived control (Pierce *et al*., 2003). Psychological ownership is composed of three motives: one’s need to experience and display effectiveness and competence, one’s need for identity and one’s need to feel a sense of belonging. In line with these motives, psychological ownership tends to develop as individuals invest themselves in the organization through investing their time, knowledge and resources into the organization, further developing a perceived sense of control over certain aspects of that organization, such that the more resources invested or aspects controlled, the stronger the sense of psychological ownership (Pierce *et al*., 2003). More recently, the concept of psychological ownership has been extended to reflect the distinction between promotive and preventative forms of psychological ownership (Avey *et al*., 2009) and the introduction of collective psychological ownership (Pierce and Jussila, 2010; for reviews, see Dawkins *et al*., 2017; Yu, 2021; for a bibliometric overview, see Kim *et al*., 2024).

Numerous studies have examined antecedents of psychological ownership (van Dyne and Pierce, 2004; for a meta-analysis, see Zhang *et al*., 2021). Consistent with the classic framework, these antecedents can be organized around three primary routes through which ownership develops (Pierce *et al*., 2001): (1) control over the target (e.g. autonomy and participation in decision-making), (2) intimate knowing of the target (e.g. information sharing and familiarity that deepen understanding of the organization) and (3) investment of the self in the target (e.g. time, effort, and personal energy devoted to one’s work and organization). Zhang *et al*.’s (2021) meta-analysis also identifies a set of relational and safety-oriented antecedents (e.g. including organizational justice, trust, perceived organizational support and relational closeness), suggesting that employees may come to feel ownership not only through direct control or personal investment, but also when the organization is experienced as fair, supportive and trustworthy. In the healthcare sector, where interdependence and uncertainty are common, these relational factors may be especially important for fostering psychological ownership.

Fostering a feeling of ownership of the organization can be very valuable, as research has identified that psychological ownership leads to positive outcomes for the employees and the organization. For instance, when an employee feels a sense of ownership within an organization, they experience a sense of responsibility and loyalty toward that organization, which results in positive behavior engagement (e.g. Avey *et al*., 2009). Psychological ownership has also been found to be positively associated with extra-role behaviors, meaning that employees who feel some sense of psychological ownership in their work tend to go above and beyond the requirements of their role (Mayhew *et al*., 2007). In a meta-analysis, Zhang *et al*. (2021) found that psychological ownership can predict organizational performance above and beyond organizational identification and organizational commitment. Another meta-analysis identified psychological ownership as a link between organizational factors and positive organizational outcomes (Renz, 2024). For instance, Renz (2024) found that factors such as work design, benefits, and communication practices influence psychological ownership, which in turn fosters positive attitudes and greater performance. Additionally, psychological ownership is negatively associated with leaving an organization, indicating its mitigating effect on employee turnover (Avey *et al*., 2009). The effect has also been found in the healthcare sector, where psychological ownership has a key role in lowering intention to leave (Sharif *et al*., 2021). This positive relationship between psychological ownership and turnover has received more attention in recent years, but has not been studied as a factor accounting for the negative association between depressive symptoms and intentions to quit.

### Work engagement

Work engagement is another critical resource associated with employee retention (Alarcon and Edwards, 2010). Engaged employees complete work duties in a timely manner and are perseverant and efficient when dealing with job demands (Schaufeli *et al*., 2002) and contribute to greater financial performance and organizational success (Bakker and Schaufeli, 2008). Experts define work engagement as a positive and fulfilling state characterized by (1) absorption, (2) vigor and (3) dedication (Schaufeli *et al*., 2002). The first characteristic, absorption (i.e. incorporation), is equivalent to losing oneself in one's work via a total focus of attention, enjoyment, control and a lack of self-consciousness (Schaufeli *et al*., 2002). Second, vigor (i.e. energy), is characterized by mental perseverance and resilience despite difficulties; it is a positive emotional state that facilitates dedication to one’s work (Schaufeli and Bakker, 2004). Lastly, dedication (i.e. commitment) is displayed through excitement, pride, challenge, inspiration and a sense of importance (Schaufeli and Bakker, 2004).

As a major component of the JD-R framework, work engagement has been the focus of a substantial amount of research, including the investigation of its antecedents that comprise individual and situational factors (Bakker *et al*., 2023). Many individual factors have been identified as predictors of work engagement, including conscientiousness, positive affect, proactive personality (Christian *et al*., 2011), self-efficacy, optimism and self-esteem (Mäkikangas *et al*., 2013). Regarding the situational factors, results from meta-analyses suggest that job resources such as autonomy, feedback or social support predict work engagement (Christian *et al*., 2011; Lesener *et al*., 2020; Mazzetti *et al*., 2023). Lesener *et al*. (2020) categorized these resources into three groups (i.e. group-level resources, leader-level resources and organizational-level resources) and found that resources from these three levels were associated with work engagement. Rather than categorizing resources by level, Mazzetti *et al*. (2023) categorized them into four types (i.e. job resources, social resources, organizational resources and developmental resources). These four types of resources were also positively associated with work engagement. Moreover, people’s feeling of engagement in their work is not static, instead it can be improved by workplace intervention, including bottom-up, resource-developing intervention (Björk *et al*., 2021; Knight *et al*., 2017).

There is also an ample amount of research that investigated the consequences of work engagement. For instance, work engagement leads to greater levels of job satisfaction (Mazzetti *et al*., 2023), commitment (Mazzetti *et al*., 2023), openness to new experiences (Fredrickson, 2001), greater creativity (Bakker *et al*., 2020) and greater levels of job performance (Neuber *et al*., 2022), and extra-role performance (Christian *et al*., 2011). In a systematic review conducted on the healthcare sector, Keyko *et al*. (2016) found that work engagement was positively associated with perceived quality of care, patient satisfaction and work effectiveness. Additionally, multiple studies have highlighted work engagement as a predictor of turnover or turnover intention, including some in the healthcare sector (e.g. Alarcon and Edwards, 2010; Bakker and Schaufeli, 2008; Mazzetti *et al*., 2023; Schaufeli *et al*., 2002; Xu *et al*., 2024; Yamamoto *et al*., 2026).

Together, psychological ownership and work engagement may account for the negative effects of depression on organizational longevity. Despite extensive research on depression among HCWs and its outcomes (Belete and Anbesaw, 2022; Fond *et al*., 2022; Jamaludin and Binti Zulkifli, 2025; Pang *et al*., 2020; Wang and Welbourne, 2024) and on drivers of employee turnover, there is a gap in the literature assessing whether these effects remain among employees experiencing MDD or depressive symptoms. Although mental health and depressive symptoms have been linked to turnover intention, many of these studies focus on the stressors that would increase turnover (Schaufeli, 2017; for an example of a study conducted in the healthcare sector, see Qin *et al*., 2023; for a meta-analysis, see Lesener *et al*., 2019), rather than the resources that would foster longevity in the organization. The JD-R framework suggests that both processes (i.e. job demands and job resources) are important and that job resources are the primary predictor of organizational commitment via the motivation path (Bakker *et al*., 2023). Therefore, when investigating longevity in an organization, we should not only focus on the demands and strains, but we should also consider the resources and motivation (Bakker and Demerouti, 2017). Thus, we consider the concepts of psychological ownership and work engagement as resources when examining the relationship between depression and organizational longevity. As such, the purpose of the present study is to fill this gap by examining the relationship between depression and organizational longevity in HCWs and to assess if psychological ownership and/or work engagement account for the relationship between depression and organizational longevity. The hypotheses are as follows:

H1.
Depressive symptoms will be negatively associated with workplace longevity after controlling for demographic variables (i.e. age and gender), such that 5-year intention to stay with the organization will be lower among individuals experiencing greater depressive symptoms and higher among those with less depressive symptomology.

H2.
Psychological ownership and work engagement will account for the negative relationship between depressive symptomology and organizational longevity after controlling for demographic variables (i.e., age and gender), such that individuals who score higher on these two factors will display higher 5-year intention to stay with the organization, regardless of depressive symptomology.

## Methods

### Participants and procedure

A cross-sectional survey was administered to 206 US healthcare workers who were recruited through Prolific, an online participant recruitment platform designed for scientific research (Palan and Schitter, 2018). Prior studies have shown that Prolific yields high-quality data comparable to, and sometimes exceeding, that obtained from other crowdsourcing platforms (Peer *et al*., 2017). The respondents were paid $6 to complete the survey. The participants provided consent, and the anonymous survey took approximately 25 min to complete. This was approved by the university’s institutional review board (IRB # 10001).

Of the 206 participants who participated in the study, 199 completed the Patient Health Questionnaire (PHQ-9), which was used to assess depression symptomology and MDD, and were included in the final analysis. The demographic makeup for the participants in this study indicated a mean age of 39.9 (*SD* = 12.1), ranging from 21 to 82 years. Further, 73.9% reported as women, 24.1% as men, 0.5% as nonbinary and 1.5% did not respond. For race, 69.3% reported being white, 19.6% black, 3.0% other/multi-race, and 8.0% declined to report race. Further, 8.5% reported being of Spanish, Hispanic or Latino origin, and 91.5% reported not being of Spanish, Hispanic, or Latino origin. For relationship status, 54.8% reported being married, 10.6% reported living with a partner, 22.6% reported being single and 12.0% reported being divorced, separated, or widowed. Based on the highest level of education attained, 6.5% completed high school or GED, 24.7% reported some college, Associate’s degree or technical degree, 35.7% had a Bachelor’s degree, and 32.7% had a graduate or professional degree. For current occupational demographics, the median number of years at current job was 6.0 (*M* = 7.1, *SD* = 6.0), the median number of hours worked per week was 40.0 (*M* = 37.2, *SD* = 9.1), and the base salary per year ranged between $3,000 and $355,200 with a median base salary of $62,500/year (*M* = $69,649, *SD* = $43,435).

### Measures

Participants were asked to provide information about their age, gender identity, race, ethnicity and marital status. Further demographic questions assessed their education level, years at current position, base salary and hours worked per week.

Depressive symptomology and Major Depression were evaluated using the Patient Health Questionnaire (PHQ-9; Kroenke *et al*., 2001). The PHQ-9 subscale includes 9 statements to which participants are asked to indicate how frequently they experienced them within the past 2 weeks using a 4-point Likert scale from 0 = Not at All to 3 = Nearly Every Day. An example of an item on this scale is: “Little interest or pleasure in doing things.” Summed scores are used to assess depressive symptomology, with higher scores indicating greater levels of depression symptoms. Scores of 10 or above are used to determine if an individual meets the criteria for the provisional diagnosis of MDD (Levis *et al*., 2019). The internal consistency for the PHQ-9 was strong (Cronbach’s alpha = 0.91).

Psychological Ownership was assessed using the Psychological Ownership Scale (Van Dyne and Pierce, 2004), which includes 7 agreement statements measured on a 5-point agreement scale from 1 = Strongly Disagree to 5 = Strongly Agree. Examples of statements from this scale are “This is MY organization” and “I feel a very high degree of personal ownership for this organization.” The internal consistency of this measure was strong (Cronbach’s alpha = 0.91).

Work Engagement was assessed using the Intellectual, Social and Affective Engagement Scale (ISA; 9-items; Soane *et al*., 2012). This assessment has three subscales: Intellectual Engagement, Social Engagement, and Affective Engagement, measured on a 7-point Likert scale from strongly disagree to strongly agree. Examples of items on the ISA are “I focus hard on my work” and “I share the same work attitudes as my colleagues.” The internal consistency of this measure was good (Cronbach’s alpha = 0.91).

Organizational Longevity was assessed using a single-item statement developed specifically for this study. Participants were asked to indicate on a visual analog scale (VAS) their response to this statement: “On a scale from 0% to 100%, what is the likelihood that you will stay at your organization for at least 5 more years?”

### Statistical analysis

The participants in the study who received a provisional diagnosis of MDD were compared to those without MDD on demographic and occupational variables using independent *t*-tests or Mann–Whitney U tests for continuous variables and Chi-square tests of independence for categorical variables. Pairwise deletion was used for any missing data points in the univariate comparisons. Next, a hierarchical linear regression model was developed to assess the relationship between depression symptomology and the likelihood of staying with the organization for at least 5 years, and how that relationship is affected by psychological ownership and work engagement. The first block of the model included depression symptomology as a predictor, while controlling for age and gender. The second block of the model included depression symptomology, psychological ownership and work engagement, while controlling for age and gender. Multicollinearity diagnostics indicated that tolerance values were all >0.20, suggesting no serious multicollinearity concerns. To address potential violations of normality assumptions and to obtain more robust estimates, all regression coefficients were evaluated using a nonparametric bootstrapping procedure with 10,000 resamples. Bias-corrected and accelerated (BCa) 95% confidence intervals were reported. SPSS version 29 (IBM, Inc., Chicago) was used for all statistical analyses.

## Results

Of the 199 participants who completed the PHQ-9, 44 (22.1%) met the criteria for a provisional diagnosis of MDD, while 155 (77.9%) did not have MDD. Univariate comparisons were conducted to assess differences between those with and without MDD on demographic and work-related factors. No differences were identified on any of the demographics (age, gender, ethnicity, race, marital status or education level), nor were any differences found between the comparison groups on work-related factors (years at current job, hours working per week or base salary; see Table 1).

**Table 1:** Demographic comparisons between healthcare workers with and without MDD

	MDD *N* = 44	No MDD *N* = 155	*p*-value
Age	38.7 (10.5)	40.3 (12.5)	0.445
Gender (% Male)	23.3% (10)	24.8% (38)	0.845
Ethnicity (% Hispanic)	11.4% (5)	7.7% (12)	0.448
Race			0.920
White	76.9% (30)	75.0% (108)	
Black	20.5% (8)	21.5% (31)	
Other race	2.6% (1)	3.5% (5)	
Marital status			0.768
Married	50.0% (22)	56.1% (87)	
Living with partner	9.1% (4)	11.0% (17)	
Widowed	4.5% (2)	4.5% (7)	
Divorced/Separated	11.4% (5)	6.5% (10)	
Never married	25.% (11)	21.0% (34	
Education level			0.244
High School/GED	2.3% (1)	7.7% (12)	
Some college	18.6% (8)	10.3% (16)	
Associates/Technical	14.0% (6)	12.3% (19)	
Bachelor’s Degree	41.9% (18)	34.2% (53)	
Graduate/Profession degree	23.3% (10)	35.5% (55)	
Years At current job	7.2 (5.9)	7.1 (6.0)	0.955
Hours working per week	38.8 (7.6)	36.7 (9.4)	0.165
Base salary	65k (35k)	71k (45k)	0.472

**Note(s):** Values shown are Means (St. Deviations) for continuous variables and Percentages (Counts) for categorical variables

When comparing occupational factors between those with and without MDD (see Table 2), there were significant differences in work engagement, such that those with MDD had significantly lower levels of engagement than those without MDD (*p* < 0.001). No differences were identified between the two MDD groups when comparing psychological ownership (*p* = 0.461). However, when comparing the MDD groups on 5-year organizational longevity, we evaluated this in two ways due to the non-normality of the distribution. First, we ran an independent *t*-test using bootstrapping, which did not show significance between group means (*p* = 0.199). We then ran a Mann–Whitney U test, which had a marginally significant difference in medians (*p* = 0.061), with the trend showing a higher likelihood of staying with the organization for those without MDD.

**Table 2:** Occupational comparisons between healthcare workers with and without MDD

	MDD *N* = 44	No MDD *N* = 155	*p*-value
Work engagement	44.4 (9.1)	51.0 (8.4)	<0.001
Psychological ownership	20.6 (7.2)	21.5 (7.2)	0.461
Organizational longevity (5 year)	60.1 (31.0)	67.4 (33.9)	0.199
	Median = 66.0	Median = 75.0	0.061

**Note(s):** Unless otherwise noted, the values shown are Means (St. Deviations)

A correlation matrix including depression symptomology, psychological ownership, work engagement and 5-year longevity is shown in Table 3. Each of the predictor variables had a significantly small-to-moderate correlation with 5-year longevity.

**Table 3:** Correlation matrix

	5-Year longevity	Depressive symptomology	Psychological ownership
5-Year longevity			
Depressive symptomology	−0.16*		
Psychological ownership	0.36**	−0.08	
Work engagement	0.29**	−0.38**	0.32**

**Note(s):** *p < 0.05, **p < 0.01

Lastly, a hierarchical linear regression model was developed to first assess the relationship between depression symptomology and 5-year organizational longevity, then to see how psychological ownership and work engagement affected that relationship (see Table 4). Block 1, which includes depression symptomology and controls for age and gender, significantly predicts 5-year longevity with a small effect, *R*^2^ = 0.03, *F*(3, 189) = 1.96, *p* = 0.121. The model shows a negative significant association between depression symptoms and 5-year longevity (β = −0.17, *p* = 0.021). In Block 2, the psychological ownership and work engagement variables are also included. Block 2 significantly predicts 5-year longevity with a moderate effect, *R*^2^ = 0.18, *F*(5, 187) = 8.31, *p* < 0.001. Work engagement’s association with 5-year longevity was small, positive and marginally significant (β = 0.15, *p* = 0.061). The relationship between psychological ownership and 5-year longevity was positive, moderate, and significant (β = 0.32, *p* < 0.001). The addition of psychological ownership and work engagement in Block 2 explained an additional 15.2% of the variance in 5-year longevity. Notably, the depressive symptomology becomes nonsignificant with the addition of psychological ownership and work engagement, indicating that these protective factors account for the negative relationship between depression and 5-year longevity.

**Table 4:** Hierarchical linear regression: assessing how depressive symptoms, worker engagement and psychological ownership relate to organizational longevity (5 years)

	B	SE (Boot)	BCa 95% CI	β	*p*-value	B	SE (Boot)	BCa 95% CI	β	*p*-value
	Block 1					Block 2				
Age	−0.19	0.24	−0.65, 0.31	−0.07	0.421	−0.15	0.24	−0.56, 0.31	−0.05	0.499
Gender (ref: Man)	0.59	5.45	−9.60, 11.26	0.01	0.919	0.25	5.45	−9.74, 10.37	0.00	0.963
Depressive symptoms	−0.88	0.38	−1.64, −0.21	−0.17	0.021	−0.44	0.38	−1.22, 0.29	−0.08	0.253
Work engagement						0.55	0.30	−0.03, 1.16	0.15	0.061
Psychological ownership						1.15	0.23	0.71, 1.57	0.32	<0.001
	Block 1					Block 2				
	*R*	*R* ^2^	Adjusted *R*^2^	F	*p*-value	*R*	*R* ^2^	Adjusted *R*^2^	F	*p*-value
Model summary	0.174	0.03	0.02	1.96	0.121	0.43	0.18	0.16	8.31	<0.001

**Note(s):** Bootstrapped standard errors and 95% confidence intervals are based on 10,000 resamples using the bias-corrected accelerated (BCa) method

## Discussion

From an organizational perspective, understanding the factors that influence workplace longevity is essential for designing effective retention strategies. Since depression has been shown to predict turnover in HCWs, it is important to explore ways that organizations can intervene to mitigate high attrition. The current study explored the relationship between depression and organizational longevity in healthcare workers and assessed the role of psychological ownership and workplace engagement in accounting for this relationship. Our study aimed to contribute to the JD-R framework by investigating whether job resources (i.e., psychological ownership and work engagement) play a role when considering the relationship between an indicator of ill-being (i.e., depression) and longevity in an organization. The most recent formulation of the JD-R framework emphasizes the dynamic aspects of the two processes (Bakker and Demerouti, 2017; Bakker *et al*., 2023). By considering psychological ownership and work engagement in addition to depression, our study examines the effects of job resources alongside the strains in a very specific environment. Thus, the purpose of this study was to explore reasons why some employees are willing to stay in their organizations despite meeting the criteria for provisional diagnosis of MDD. In addition, while the concept of work engagement is essential to the JD-R theory, the concept of psychological ownership is less central (Bakker and Demerouti, 2017; Bakker *et al*., 2023). With the inclusion of psychological ownership besides work engagement, we aim to add to the JD-R framework by including in the definition of resources, the strong sense of connection with the organization (this organization is “mine”). Our findings support Hypothesis 1 and indicate that HCWs experiencing MDD are significantly less likely to stay with the organization for 5 years when compared to those without MDD. These findings are in line with recent research showing that psychological distress is associated with negative organizational outcomes, including lower retention (Almeida-Meza *et al*., 2025; Jachmann *et al*., 2025; Zhang *et al*., 2024). This is a major issue that concerns roughly one-third of HCWs (Fond *et al*., 2022).

Additionally, our findings support Hypothesis 2 by indicating that psychological ownership and work engagement do account for the negative relationship between MDD and organizational longevity, such that the negative association between MDD and organizational longevity becomes nonsignificant when psychological ownership and work engagement are included. These results are in line with previous research that found that psychological ownership had a mitigating effect on employee turnover (Avey *et al*., 2009; Sharif *et al*., 2021). Similarly, these results are consistent with research on work engagement that found that work engagement was an effective predictor of turnover or turnover intention (e.g. Mazzetti *et al*., 2023; Xu *et al*., 2024; Yamamoto *et al*., 2026). Overall, these findings align with the JD-R theory, which describes that when people have more resources, such as psychological ownership and work engagement, they cope better with the demands (Bakker and Demerouti, 2017; Bakker *et al*., 2023). Thus, these resources can support employees who meet the criteria for a provisional diagnosis of MDD to envision staying longer in their organization. Our results are consistent with prior studies demonstrating the negative impact of depression on turnover intention (Fond *et al*., 2022; Olaya *et al*., 2021; Pang *et al*., 2020) and the role of psychological ownership and engagement in accounting for turnover (Alarcon and Edwards, 2010; Avey *et al*., 2009; Bakker and Schaufeli, 2008; Mayhew *et al*., 2007). However, it is paramount to ensure that the onus is not only on the individuals to find ways to cope better with stressful situations and depression, but it is also on organizations to provide a place where HCWs feel safe to work and to thrive. Shifts in organizational priorities and practices (Amoadu *et al*., 2023), changes in work design (van Kraaij *et al*., 2025), as well as interventions (Aust *et al*., 2024), can help HCWs cope better with the stressors. By integrating these literatures, our study helps fill an important gap regarding turnover intention in HCWs experiencing clinically significant depressive symptoms and opens avenues for further inquiry into resource-based interventions across diverse industries.

Moreover, most applications of the JD-R framework investigating turnover intention in the healthcare sector have focused on the demands (Lesener *et al*., 2019; Qin *et al*., 2023; Schaufeli, 2017). However, turnover intention and intention to stay are influenced by numerous factors (Wynendaele *et al*., 2025). Our study not only considers demands, but also resources, and by showing that depressive symptoms are not a significant predictor of 5-year intention to stay once psychological ownership and work engagement are included, our results suggest that resources and the resource-based process also have an impact on longevity in the organization. These findings are in line with more recent developments of the JD-R theory that stipulates links between the two processes (Bakker and Demerouti, 2017; Bakker *et al*., 2023).

### Strengths and limitations

This study had several strengths and limitations. The demographic breakdown of our sample includes around 70% female participants, which reflects the gender split of all HCWs, speaking to the strength of the study’s generalizability (Ghebreyesus, 2019). To further strengthen generalizability, we collected data from a wide and representative age range.

Moreover, our study measured organizational longevity, which aligns with a greater emphasis in recent years on considering intent to stay (e.g. Hendrickson *et al*., 2022; Sánchez-Cardona *et al.*, 2023). Our study is not without limitations. First, the demographic breakdown of our sample was majority white HCWs, which may not be representative of the HCW population in the United States. We did not collect data with regard to how long our participants have been working in healthcare, which could potentially be a mitigating factor with regard to employee outcomes (An *et al*., 2022). We used a one-item measure of organizational longevity, which limits the assessment of nuanced feelings about intention to stay and or to leave the organization. Future studies could use different measures of intention to stay and to leave, as it will paint a richer picture of how job demands and job resources influence these feelings. Furthermore, interviews could also shed light on how people feel about these demands and resources and their longevity in the organization. Additionally, we did not collect data with regard to any comorbidities among those who were classified with MDD, which could have been contributors to the higher levels of intention to leave. Our sample was relatively small, and all data collected were cross-sectional and self-reported, which prevents us from making causal inferences (Antonakis *et al*., 2010) and can increase the risk for common method bias (Podsakoff *et al*., 2024). Future studies should use longitudinal designs to test causal associations. Finally, future studies could consider other resources (e.g. psychosocial safety climate; Amoadu *et al.*, 2025; Karatuna *et al*., 2025) that could potentially mitigate the impact of job demands on longevity in the organization.

### Implications

The findings from this study are particularly relevant given the ongoing global shortage of HCWs. From an organizational perspective, it is important to investigate the workplace correlates of workplace longevity. Possessing this information allows for developing our understanding of why people intend to stay or to leave their jobs, which could contribute to identifying factors that promote or prevent people from staying in their organization. Down the line, this could help with the development and implementation of interventions that can improve retention within the healthcare industry. Specifically, our study found that allowing employees more psychological ownership of and engagement with their work could potentially be effective in counteracting the effects of depression on employee turnover. Thus, organizations should create conditions that allow HCWs to develop their resources. For example, to strengthen the feelings of psychological ownership, they could promote organizational justice, organizational support and trust (Zhang *et al*., 2021). Furthermore, to cultivate work engagement, organizations could provide rich feedback and enable employees to craft their job (Bakker and Demerouti, 2017). This would not only be beneficial for employees, but it would also be beneficial for organizations (Razmpour *et al*., 2026).

Our study also has societal implications as there is evidence that higher turnover among nurses and physicians is associated with adverse patient outcomes (e.g. patient falls, mortality risks; Mauricio *et al*., 2025; Moscelli *et al*., 2024). High turnover also has implications for staffing shortages (Chang *et al*., 2025), which may affect surge capacity and resilience of the healthcare system (Leuchter *et al*., 2025). Thus, our findings support recent research that calls for systemic implementation/organizational accountability and policy-relevant actions in the healthcare sector (Frias *et al*., 2025). For instance, healthcare organizations could use psychosocial safety climate as a system-level indicator of HCWs mental health and safety (Amoadu *et al.*, 2025) and aim not only to reduce turnover but also to increase long-term retention and staffing stability (Chang *et al*., 2025).

## Conclusion

Our study sought to investigate the association between depression and worker longevity among HCWs, and to identify whether work engagement and psychological ownership account for that relationship. Our findings support the notion that work engagement and psychological ownership significantly impact the negative relationship between depression and HCW longevity. High turnover in the healthcare industry is costly to the organization, the employees and the patients. The results from this study show that promoting employee psychological ownership initiatives and strengthening work engagement could be beneficial for employee retention. This research helps address the gap in turnover intention in HCWs experiencing depression, and hopefully inspires future work on factors that can improve workplace longevity in HCWs with MDD.
